# Berlin and Epworth Surveys to Predict Obstructive Sleep Apnea for Adults on Biomimetic Oral Appliance Therapy: A Nonrandomized Clinical Trial

**DOI:** 10.1155/2022/5283406

**Published:** 2022-05-06

**Authors:** Noor Al Mortadi, Basheer Khassawneh, Lina Khasawneh, Osama Alzoubi, Karem H. Alzoubi

**Affiliations:** ^1^Department of Applied Dental Sciences, Faculty of Applied Medical Sciences, Jordan University of Science and Technology, Irbid, Jordan; ^2^Department of Internal Medicine, Faculty of Medicine, Jordan University of Science and Technology, Irbid, Jordan; ^3^Department of Prosthodontics, Faculty of Dentistry, Jordan University of Science and Technology, Irbid, Jordan; ^4^School of Medicine, The University of Jordan, Amman, Jordan; ^5^Department of Pharmacy Practice and Pharmacotherapeutics, University of Sharjah, Sharjah, UAE; ^6^Department of Clinical Pharmacy, Jordan University of Science and Technology, Irbid, Jordan

## Abstract

**Background:**

Two questionnaires (Berlin Questionnaire (BQ) and Epworth Sleepiness Scale (ESS)) are the widely used screening instruments for subjects suffering from sleep disorders. Obstructive sleep apnea (OSA) is the most common form of sleep-disordered breathing. The biomimetic oral appliance therapy (BOAT) offers an alternative nonsurgical method, which can improve symptoms and indices of OSA on objective sleep testing.

**Aim:**

To describe testing the ability of BQ and EES for prediction of BOAT outcomes during OSA.

**Methods:**

Seventeen adults (9 males, 8 females; age, mean (SD): 45.76 (10.31), BMI mean (SD): 33.5(13.43)) who underwent an overnight sleep study were diagnosed by a sleep specialist physician. The BQ and EES were recorded before and after BOAT treatment. Subjects with mild-to-moderate OSA had 2 months of follow-up visits and underwent a final overnight sleep study to measure apnea-hypopnea index (AHI). The subjects were asked to wear the appliance for 10–12 hours/day and at night. Findings were analyzed statistically using paired *t*-tests.

**Result:**

As per sleep test results, pre-BOAT AHI measures versus post-BOAT AHI measures showed significant improvement. Comparing the BQ before versus after treatment showed that at the pretreatment stage, 66.0% of patients had high-risk score, whereas 34% had low-risk score. After treatment, 66.0% of patient had low-risk scores, whereas 34% had high-risk scores. As for the ESS, treatment resulted in significant reduction of total score from 10.43 ± 6.32 to 5.00 ± 5.20 (*P* < 0.01, paired *t*-test). Finally, there was a mild negative correlation between AHI and each of the BQ and ESS scores that was not statistically significant (*r* = −0.420, *N* = 26, *P* > 0.05, and *r* = −0.41, *N* = 26, *P* > 0.05, respectively).

**Conclusion:**

The BOAT device may provide a useful form of therapy to improve OSA-related PSG parameters such as AHI. Both BQ and ESS were predictive to improvements detected by the sleep study during BOAT device use.

## 1. Introduction

Obstructive sleep apnea (OSA) is an increasingly common sleep-disordered breathing (SDB) condition [1]. It is characterized by repeated episodes of partial or complete obstruction of the respiratory passages during sleep [2–4]. Daytime sleepiness, insomnia, nocturia, and morning headaches are symptoms of OSA [5, 6].

In-laboratory polysomnography (PSG) is the gold standard diagnostic test for OSA, whereas the severity of the condition is usually assessed on the basis of the apnea-hypopnea index (AHI). The AHI describes mild, moderate, or severe OSA corresponding to AHI between 5–14, 15–30, and greater than 30 events/h, respectively [7]. In the clinical setting, there are many sleep questionnaires used to screen for and assess OSA symptoms. These tools are rarely—if ever—used by sleep experts and should never replace PSG, as they have variable diagnostic and risk stratification performances [8]. The Berlin Questionnaire (BQ) consists of 10 items divided into three categories: relating to snoring and witnessed apneas (5 items), daytime sleepiness (4 items), and hypertension/BMI (one item) [9]. The score stratifies patients as having a high or low risk for OSA, in which studies have demonstrated 80% sensitivity and 46% specificity to detect OSA when it is defined as a AHI of 5–14 events/h. The BQ also showed 91% sensitivity and 37% specificity to detect OSA when defined as a AHI of ≥15 events/h [10]. Despite that, the American Academy of Sleep Medicine (AASM) recommended against using such questionnaires to screen asymptomatic individuals for OSA, as diagnostic accuracy values of such tools are mostly derived from referral-based populations which do not reflect the general population [8].

The Epworth Sleepiness Scale (ESS) is another tool to assess the level of daytime sleepiness by asking the subject to rate on a scale of 0 to 3, on the chances that he would doze in each of eight different situations [11]. An ESS score of more than 10 indicates abnormal sleepiness and should prompt further testing [11].

The prevalence of OSA is variable and depends on an individual's age, gender, nationality, and BMI, as well as on the methodology of the criteria used for diagnosis. The prevalence of OSA in the general population ranges from 2% to 32.8% [12, 13]. A recent systematic review of epidemiological studies across different geographic locations revealed that the prevalence of OSA ranged from 9% up to 38% and was consistently higher in men [14]. Obesity is recognized as a major risk factor for OSA, which has been increasing worldwide throughout the past decades [15, 16]. A cross-sectional survey in Jordan utilized the BQ to quantify the prevalence of individuals with high risk of OSA in primary care which was 16.8% [17]. Frequent daytime fatigue or tiredness was present in 33.9%, while snoring was present in 28.7% [17].

Continuous positive airway pressure (CPAP) is the mainstay of therapy for adults with OSA and involves sleeping while breathing air that is at increased pressures (as compared to the ambient atmospheric pressure) through different designs and types of masks [18]. CPAP, thus, maintains a positive pharyngeal transmural pressure so that the intraluminal pressure exceeds the surrounding pressure and stabilizes the upper airway thus preventing respiratory events via upper airway collapse (e.g., apneas and hypopneas) [19, 20].

Oral appliance therapy is a reasonable alternative treatment for patients with mild or moderate OSA, who decline or fail to adhere to continuous positive airway pressure (CPAP) [21]. Mandibular advancement devices (MADs) are the typical and most common oral appliances for OSA [22], which are anchored to the teeth and induce mandibular advancement, thus mechanically enlarging the airway while being worn during sleep [22, 23]. Biomimetic oral appliance therapy (BOAT) is a type of oral appliances that, unlike the conventional MADs, aims to mimic natural craniofacial growth and development to induce upper airway remodeling that persists even after removal of the device [23]. BOAT has been shown to increase the nasal cavity volume and many craniofacial parameters [24, 25]. The aim of the current study is to investigate the sensitivity of the Epworth and Berlin questionnaires to predict the outcomes of the use of the novel protocol of BOAT, Daytime-Nighttime Appliance (the DNA appliance® system), as a treatment tool for OSA. Thus, the statistical null hypothesis of the current study is that Epworth and Berlin questionnaires cannot predict the outcomes of the use of the novel protocol of BOAT among OSA patients.

## 2. Method

### 2.1. Selection of the Subjects

Patient recruitment was based on referrals from the sleep-medicine clinic at the King Abdullah University Hospital (KAUH) in Jordan. Medical records of 150 patients were carefully screened followed by interviewing fifty candidate patients individually. Twenty-seven patients were chosen according to the inclusion and exclusion criteria in a dental clinic. Seventeen patients committed to study appointments and were included in our final analysis.

The inclusion criteria encompassed patients above 18 years of age with a confirmed diagnosis of mild-to-moderate OSA by PSG or severe OSA and intolerant or refused CPAP therapy. Patients also should have good oral hygiene with enough number of maxillary and mandibular teeth to retain the removable device. Excluded were patients who were unable to attend regular appointments. The participants were recruited after signing informed consent of the study. The study protocol was reviewed and approved by the institutional review board at Jordan University of Science and Technology (JUST) (IRB number is 15/108/2017, https://ClinicalTrials.gov identifier: https://clinicaltrials.gov/ct2/show/NCT05087316). The study subjects were protected by following the Declaration of Helsinki and its amendments. The classification of OSA severity was based on AHI (mild defined as an AHI of 5–14, moderate as 15–30, and severe as >30) [7]. The sample size of the study was calculated using G-Power 3.1., Universitat Kiel, Germany, based on the convenience sample method, large effect size, alpha value of 0.05, and power of 0.80. The required minimum number of subjects was 17.

### 2.2. Berlin and Epworth Sleepiness Scale Questionnaires

The patients were interviewed for Berlin and Epworth Sleepiness Scale questionnaires. The two questionnaires were filled out by the researchers and double-checked with patients. Data regarding gender, age, height, weight, and race were also obtained. The Berlin Questionnaire was used to identify patients as being at “high” or “low” risk for obstructive sleep apnea based on their responses for each category of items [9]. On the other hand, Epworth Sleepiness Scale (ESS) Questionnaire was used to assess the sleepiness of patients, in which patients with scores above 10 were considered as having significant daytime sleepiness and scores above 15 were considered to have pathological sleepiness.

### 2.3. DNA Appliance™

**Figure 1:**
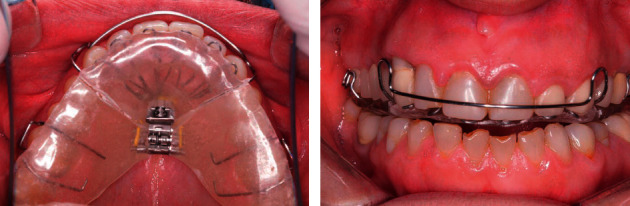
The acrylic-based DNA appliance® used in this study showing 6 (patented) anterior 3-D axial springs®, a midline screw, posterior occlusal coverage, retentive clasps, and a labial bow. Note: other designs are customized depending on patient presentation.

Patients were given Daytime-Nighttime Appliance (the DNA appliance® system, BioModeling Solutions, Inc., Beaverton, OR, USA). After careful history-taking and craniofacial examination, a bite registration was obtained in the upright-sitting position with corrected jaw posture in the vertical axis specific for each subject. Upper and lower jaws were mounted using the bite registration, and the DNA appliance™ system were custom-fabricated for each subject in the dental laboratory and delivered by a dentist. Subjects were asked to wear the appliances for a period of 10–12 hours in the evening and at night. Participants were instructed that the appliance should not be used during the day or while eating, partly in line with the circadian rhythm of tooth eruption [21]. Written and verbal instructions were given to all subjects. The DNA appliance is designed to correct maxillo-mandibular underdevelopment in both children and adults [26]. Typically, the DNA appliance consists of 6 patented, anterior 3-D Axial Springs™, a midline actuator (such as omega loops or screws), posterior occlusal rests, and a round labial bow (Figure 1). Patients were trained on insertion and removal of the appliance as well as on screw activation.

At the diagnosis stage and upon insertion of the devices, appliances were checked for accuracy, ensuring snug fit and expansion of the palatal screw. Only gentle pressures were transmitted to the teeth and surrounding tissues, and the functionality of the device was checked with the subject activating a mild force on biting.

### 2.4. Follow-Up Visits

The posttreatment PSG tests were accomplished with no appliance in the mouth and were monitored by a sleep physician. The mean AHI of the study sample was calculated and compared to their values at diagnosis stage.

### 2.5. Data Analysis

The results were subjected to statistical analysis (SPSS version 23, SPSS Inc, Chicago, IL, USA). Data were analyzed using the paired or unpaired *t*-test. Pearson's correlations were carried out. *P* < 0.05 was considered significant. Quantitative variables were expressed as the mean (SD).

## 3. Results

The final sample of the study consisted of 17 patients who were committed to the use of the BOAT device, of which 9 were males. The mean age of the study subjects was 45.76 (10.31), while the mean body mass index (BMI) was 33.5 (13.43). The average duration of AHI treatment was 15.4 ± 7.1 months. The OSA severity of the included patients as per the PSG test at the sleep lab was as follows: mild (AHI <15, *n* = 6, 35.3%), moderate (AHI 15–30, *n* = 8, 47.1%), and severe (AHI ≥30, *n* = 3, 17.5%).

Regarding PSG variables, pre-BOAT AHI measurements versus post-BOAT AHI measurements showed significant improvement where total AHI/hr was reduced from 26.35 ± 10.71 to 11.81 ± 10.14 (*P*=0.019). Comparison of the BQ results before and after treatment showed that at the pretreatment stage, 66.0% of patients had high-risk scores for OSA, whereas 34% had low-risk scores. However, at the posttreatment stage, 66.0% of patients were at low risk for OSA and 34% had high-risk scores. As for the ESS questionnaire, BOAT treatment resulted in a significant reduction of the total score from 10.43 (6.32) to 5.00 (5.20) (*P* < 0.01, paired *t*-test). The distribution of scores as per diagnostic criteria of the Epworth Sleepiness Scale is shown in Table 1.

Pearson's correlation was performed to determine the correlation between AHI and each of the BQ and ESS scores. There was a mild negative, but not statistically significant, correlation between AHI and each of the BQ and ESS scores (*r* = −0.420, *N* = 26, *P* > 0.05, and *r* = −0.41, *N* = 26, *P* > 0.05, respectively) (Table 2).

## 4. Discussion

This study reports two main findings. Firstly, there was a significant difference in the results of objective PSG total AHI and subjective BQ and ESS scores when patients were compared before and after BOAT treatment. This adds to the accumulative evidence of the capability of such intervention to improve OSA. Secondly, we found a mild negative correlation between AHI and each of the BQ and ESS scores that was not statistically significant, which is in concordance with the American Academy of Sleep Medicine (AASM) guidelines and sleep-medicine experts' opinions that discourage the use of such tools and questionnaires to diagnose or risk-stratify OSA patients [8].

Typical oral appliances, MADs being the most popular, exert their effect by repositioning the tongue and the mandible forward and downward to reduce airway collapse and widen the lateral aspects of the pharyngeal walls, thus improving the airway patency [27]. Mandibular protrusion is an important element of MADs action, and it is reported that effective degrees of advancement range from 6 to 10 mm or from 65% to 70% of maximum protrusion [28].

On the other hand, the rationale of using biomimetic oral appliance therapy (BOAT) is based on the idea that the upper airway is a complex adaptive system, which can undergo remodeling in pathologic conditions. It is conceptually based on the epigenetic premise that the potential for craniofacial growth and development remains intact within an individual, which can be accomplished with such oral appliances [29]. BOATs have been shown to successfully increase the nasal cavity volume, trans palatal width, and maxillary sinus volume.

Oral appliances were shown to be more preferable to patients over CPAP [30, 31], which plays a role in the observed lower rates of nonadherence which is crucial as the effectiveness of treatment is dependent on regular and prolonged use of the device [31–33]. The most frequent reasons why patients discontinued MAD use were discomfort or that the MAD had no effect [34]. Other side effects of customized MAD use included dry mouth, tooth pain, jaw discomfort, and temporomandibular joint symptoms [34]. Further exploration of patients' perspectives and experiences during applying BOAT would be of great value. For instance, a pain index such as the visual analogue score (VAS) [35, 36] can be used to evaluate tooth pain experienced when applying BOAT, which will help determine factors associated with such undesirable experiences, thus decreasing the discontinuation rates of BOATs.

Regarding the efficacy and outcomes of oral appliances to treat OSA, several studies have demonstrated how oral appliances improve objective PSG-based sleep indices, including AHI and arousal index results which were significantly lower among patients treated with oral appliances [37–40] and minimal oxygen saturation levels which were significantly higher among patients treated with oral appliances [37], among other indices, when compared to the placebo groups. These improvements appear to sustain over time [39, 40]. Upon comparison with CPAP, studies have demonstrated that both interventions are significantly effective, but CPAP is more efficacious in terms of improving AHI and oxygen desaturation indices [32, 41], while their effects on other variables such as the sleep architecture and arousal index may not be significantly different [32].

In addition to objective variables, studies have illustrated the capability of oral appliances to improve patient-centered subjective outcomes such as sleepiness and quality of life when compared to placebo [42, 43] and have found similar performances of oral appliances and CPAP in terms of these patient-centered outcomes [32, 44]. Despite some results that indicate an insignificant effect of oral appliances on these quality of life-related parameters [38], the majority of the literature including meta-analyses point toward an effective role of oral appliances in this aspect [22, 42].

Our findings regarding the significant improvement in the objective PSG-based parameter—total AHI—when comparing pretreatment baseline values with posttreatment values support the previously mentioned studies, highlighting the efficacy of oral appliances to improve AHI and desaturation indices, among other parameters. In addition, the significant differences in the ESS scores and the change in the percentages of the BQ responses between the pretreatment and posttreatment timeframes may support the ability of oral appliances to improve patient-centered outcomes such as sleepiness.

Finally, we found a mild negative correlation between AHI and each of the BQ and ESS scores that was not statistically significant, which agrees with the American Academy of Sleep Medicine (AASM) recommendations which discourage the use of such tools and questionnaires to diagnose or risk-stratify OSA patients [8].

## 5. Conclusion

The BOAT device may provide a useful form of therapy to improve OSA-related PSG parameters such as AHI and patient-centered outcomes such as sleepiness. Both BQ and ESS were predictive to improvements detected by the sleep study during BOAT device use.

## Figures and Tables

**Table 1:** Scores distribution of Epworth Sleepiness Scale before and after BOAT treatment.

	Before treatment, *N* (%)	After treatment, *N* (%)
Normal (scores 1–10)	9 (60.0%)	13 (86.7%)
Daytime sleepiness (scores >10–15)	3 (20.0%)	1 (6.7%)
Pathological sleepiness (scores >15)	3 (20.0%)	1 (6.7%)

**Table 2:** Correlations between total AHI/hr and the BQ and the ESS scores.

Correlation	AHI (r)	AHI (before)	AHI (after)
Berlin	−0.420		
Epworth	−0.418		
Berlin (before)		−0.389	
Berlin (after)			−0.356
Epworth (before)		−0.145	
Epworth (after)			−0.478

## Data Availability

Data will be available upon request to the corresponding author via e-mail.
